# A Nomogram Model Involving Immunohistochemical Markers for Predicting the Recurrence of Stage I-II Endometrial Cancer

**DOI:** 10.3389/fonc.2020.586081

**Published:** 2021-01-22

**Authors:** Peng Jiang, Mingzhu Jia, Jing Hu, Zhen Huang, Ying Deng, Zhuoying Hu

**Affiliations:** Department of Gynecology, The First Affiliated Hospital of Chongqing Medical University, Chongqing, China

**Keywords:** nomogram model, endometrial cancer, recurrence, immunohistochemical markers, classical parameters

## Abstract

**Background:**

The purpose of this study was to establish a nomogram combining classical parameters and immunohistochemical markers to predict the recurrence of patients with stage I-II endometrial cancer (EC).

**Methods:**

419 patients with stage I-II endometrial cancer who received primary surgical treatment at the First Affiliated Hospital of Chongqing Medical University were involved in this study as a training cohort. Univariate and multivariate Cox regression analysis of screening prognostic factors were performed in the training cohort to develop a nomogram model, which was further validated in 248 patients (validation cohort) from the Second Affiliated Hospital of Chongqing Medical University. The calibration curve was used for internal and external verification of the model, and the C-index was used for comparison among different models.

**Results:**

There were 51 recurrent cases in the training cohort while 31 cases in the validation cohort. Univariate analysis showed that age, histological type, histological grade, myometrial invasion, cervical stromal invasion, postoperative adjuvant treatment, and four immunohistochemical makers (Ki67, estrogen receptor, progesterone receptor, P53) were the related factors for recurrence of EC. Multivariate analysis demonstrated that histological type (P = 0.029), myometrial invasion (P = 0.003), cervical stromal invasion (P = 0.001), Ki67 (P < 0.001), ER (P = 0.009) and P53 expression (P = 0.041) were statistically correlated with recurrence of EC. Recurrence-free survival was better predicted by the proposed nomogram with a C-index of 0.832 (95% CI, 0.752–0.912) in the training cohort, and the validation set confirmed the finding with a C-index of 0.861 (95% CI, 0.755–0.967).

**Conclusion:**

The nomogram model combining classical parameters and immunohistochemical markers can better predict the recurrence in patients with FIGO stage I-II EC.

## Introduction

Endometrial cancer (EC) is a common malignant tumor in gynecology. The five-year overall survival (OS) rate is about 80%, because the majority of the patients are diagnosed at an early stage (80% in stage I) (1). However, the 5-year overall survival rate of endometrial cancer varies greatly in different stages: 50–65% for stage III and 20–25% for stage IV (2); while for stage I-II, only about 15% of patients would relapse because the lesion is limited to the uterus (3), so the 5-year OS of them is high (75–90%). Even so, relapse is still one of the leading causes of death of EC patients (4).

At present, most of the current models for predicting the recurrence of EC are based on classical clinicopathological parameters (5). For example, Lobna Ouldamer developed a nomogram model based on age, FIGO stage, histological type and grade, lymphatic vessel space invasion (LVSI), and surgical nodal staging to predict poor prognosis of patients with stage I~III EC (4); Recently, Kenta Takahashi et al. established a scoring system to assess the risk of recurrence of stage I-II EC based on age, pathological type, cervical stromal invasion, peritoneal cytology (6). However, research proves that such models can still be optimized by adding other predictive indicators (7).

Recent years, immunohistochemical markers have been widely used in clinical practice. In breast cancer, the cut-off value of the immunohistochemical marker Ki67 has become one of the important prognostic indicators (8), and recently a scoring system combining ER, PR, HER2, and Ki67 has been developed as an auxiliary tool for clinical prognosis management of breast cancer (9). Similarly, in endometrial cancer, immunohistochemical markers Ki67, ER, PR and P53 are also commonly used as prognostic indicators (10). For example, Varol Gülseren recently proposed to use the combined ratio of the two groups of the immunohistochemical markers ([P53 + Ki67]/[ER + PR]) to predict the lymph node metastasis of endometrial cancer (11); Louis J.M. suggested that adding estrogen receptor, progesterone receptor, and L1 cell adhesion molecule expression to the histology-based endometrial cancer recurrence prediction models (7). However, at present, models involving the immunohistochemical makers for predicting the recurrence of endometrial cancer are still very rare (7, 12). In this study, we developed a model to predicting the recurrence-free survival (RFS) in FIGO stage I-II EC with a combination of conventional clinicopathological factors and immunohistochemical markers.

## Material and Methods

### Study Population

This retrospective cohort study was approved by the Ethics Committee of Chongqing Medical University. The data of patients with endometrial cancer who underwent primary surgical treatment between October 2013 and May 2018 at the First Affiliated Hospital of Chongqing Medical University and the Second Affiliated Hospital of Chongqing Medical University were retrospectively analyzed. The inclusion criteria were as follows: 1. Patients diagnosed with stages I–II EC according to the International Federation of Gynecology and Obstetrics (FIGO) 2009 guidelines (13); 2. Patients with complete case record including age, body mass index (BMI), comorbidities (hypertension or diabetes), surgical procedures, pathological results (histologic type and grade, depth of myometrial invasion, cervical stromal invasion), four immunohistochemical makers (Ki67, ER, PR, P53), postoperative adjuvant treatment; while the exclusion criteria were as follows: 1. Patients did not follow the standard surgical treatment (4); 2. Pathological analysis indicated endometrial sarcoma; 3. Patients with other malignancies; 4. Patients received chemotherapy or radiotherapy before surgery; 5. Patients without regular follow-up (14).

### Treatment and Follow-Up

All patients underwent at least abdominal total hysterectomy and bilateral salpingo-oophorectomy, with or without nodal staging (sentinel lymph node ± pelvic±-para-aortic lymphadenectomy) (4), then supplemental radiotherapy or even combined with chemotherapy would be considered when patients accompany with the following high-risk factors: poor pathological classification, older age (especially >60 yrs), extensive LVSI, and deeper myoinvasion (>50%), cervical stromal invasion, tumor diameter >2 cm, etc. The specific regimens and cycles of radiotherapy and chemotherapy were determined by multidisciplinary discussion and international guidelines (15, 16). Radiotherapy was usually applied within three months after surgery (most patients’ vaginal stump wounds had healed well at this time); chemotherapy was generally started within two weeks after surgery if the patients recovered well after surgery, the interval between each chemotherapy cycle was 21 days.

Follow-up visits were performed every 3 months for the first 2 years, every 6 months for the following 3 years, and once a year thereafter. All the patients were followed up from the day of surgery onwards, follow up care plans included regular physical examinations and necessary auxiliary checks (17). Because most relapsed patients were concentrated within two years after surgery, except for a very few patients who died due to relapse or other diseases during follow-up, the remaining patients have been followed up for more than two years. The follow-up deadline was June 2020.

### Recurrence

Recurrence was considered if lesions were confirmed by physical examination, histological examination, or images, including CT, MRI, ultrasonography, bone scintigraphy, FDG-PET or specific X-ray (17). The locoregional recurrence was defined as vaginal or intrapelvic recurrence, while distant recurrence included upper para-aortic lymph node metastasis, abdominal metastasis, and metastasis to other organs (6). The recurrence-free survival (RFS) was defined as the time between date of complete surgical removal of the malignancy and either date of (histological or radiological confirmed) recurrence (18). The overall survival (OS) was defined as time from primary surgery to death as a result of any cause (17).

### Histology

All postoperative specimens were processed with the same standard in the department of Pathology (19). Briefly, samples were made into formalin-fixed, paraffin-embedded specimens. H&E staining was used to confirm which parts were cancerous. The histological type, histological grade, lesion size, lesion infiltration range were initially judged by the primary pathologist of the center, and reviewed by the superior physician. The histological types was difined as follows: type I was defined as G1 and G2 endometrioid adenocarcinoma, while the type II was defined as G3 endometrioid adenocarcinoma and non-endometrioid adenocarcinoma including serous carcinoma, clear cell carcinoma and other histotypes (6).

### Immunohistochemistry

The immunohistochemistry of ER, PR, Ki57 and P53 was performed on an automated immunostainer(Leica Bond-Max, Milton Keynes, UK). The immunohistochemical results of ER, PR, Ki57 and P53 were independently evaluated by two experienced pathologists at first, and then recorded as the percentage of positively stained tumor cells (0–100%). Pathologists’assessment for the proportion of positive tumor cells were considered to be consistent if the proportion differed no more than 10%; if the initial assessment of the proportion differed more than 10%, then the results were re-evaluated(unblinded) and a consensus reached. The two pathologists’ results for proportion were finally averaged to represent the final result of the proportion of positive tumor cells (20).

At present, many studies have shown that the decreased expression of ER and PR, the increased expression of Ki67 index were related to the poor prognosis of endometrial cancer (21), but ER, PR and Ki67 all lack recognized thresholds of positive ratios in endometrial cancer, and related literature reported that dichotomous ER and PR status offered no additional prognostic value to established clinicopathologic prognostic factors (20), so in this study, the results of ER, PR and Ki67 were expressed as continuous variables (percentage of positive rate 0–100%) instead of dichotomous variables(positive or negative).

According to the 3-tier system for P53 immunohistochemistry interpretation (22): the overexpression (the proportion of positive tumor cells ≥75%) and complete absence(the proportion of positive tumor cells was 0%) both considered as abnormal (aberrant/mutation-type), in contrast to the normal (wild-type) pattern with p53 expression levels in between these extremes (0–75%).

### Statistical Analysis

Patients from the First Affiliated Hospital of Chongqing Medical University were used to construct the prediction model. Another group of patients from the Second Affiliated Hospital of Chongqing Medical University were collected as external validation cohort. The balance and consistency between training cohort(n=419) and validation cohort(n=248) were tested: categorical data were analyzed using chi-square test and Fisher’s exact test; continuous data were analyzed using Student’s t-test and rank sum test. P values less than 0.05 were considered as statistically significant different.

In the training set, all the factors were put into the univariate Cox regression to analyze their correlation with recurrence of EC. Then the factors with P value less than 0.05 were included in the multivariate Cox regression and hazard ratios of each factors were calculated. Through the multivariate Cox regression analysis, the factors with P value less than 0.05 were selected to develop a nomogram model by R software. The Receiver Operating Characteristic (ROC) curve and the Youden Index(Youden index = sensitivity + specificity -1) were used to find an optimal threshold of the 3-year recurrence-free survival rate calculated by the nomogram (23). According to the optimal threshold, the patients in validation set were further divided into high-RFS group and low-RFS group. Then Kaplan-Meier analysis was used to describe the distribution of RFS and OS in two groups, and the log-rank test was used to compare the difference of RFS and OS between two groups.

Validation of the model was performed on the validation cohort. Firstly, performance of the predicting model was evaluated by calibration curve in both data sets respectively. The concordance index (C-index) was mainly used to evaluate the predictive performance of a model (24). To further test the efficacy of the combination of conventional clinicopathological parameters and immunohistochemical markers, the C-index for several models were calculated. Data were statistically analyzed using SPSS software (version 25.0, IBM statistics, Chicago, IL, USA) and R software (version 3.6.1, http://www.r-project.org).

## Results

**Figure 1:**
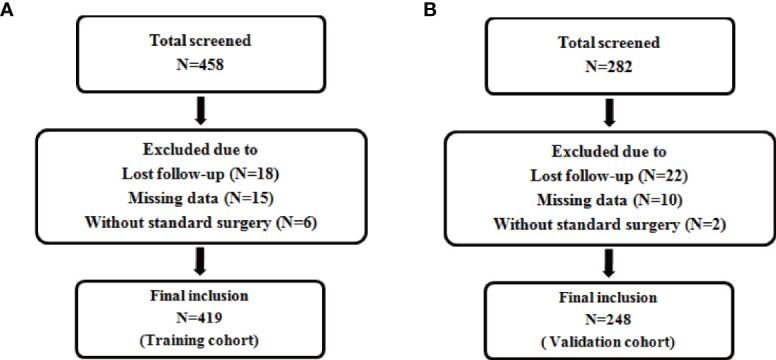
Flow chart for patient inclusion. Description: **(A)** Flow chart for inclusion of patients in the training cohort. **(B)** Flow chart for inclusion of patients in the validation cohort.

According to the inclusion and exclusion criteria, a total of 419 patients were included in the training cohort (patients were from the First Affiliated Hospital of Chongqing Medical University), and 248 patients were included in the validation cohort(patients were from the Second Affiliated Hospital of Chongqing Medical University) (**Figure 1**). The demographics and clinicopathological characteristics of women in the two cohorts were comparable (P>0.05) and were summarized in **Table 1**. A total of 51 patients relapsed in training cohort, of which 26 died due to recurrence of EC, while 31 patients relapsed in validation cohort and 18 died due to recurrence. The median follow-up were 50 (range, 12–79) months for the training cohort; as well as 47 (range, 14–79) months for the validation cohort. The clinical characteristics of relapsed patients in the two data sets were summarized in **Table 2**.

**Table 1:** Baseline characteristics of the training cohort and the validation cohort.

Variable	Training cohort N = 419	%	Validation cohort N = 248	%	P value
Age median(yrs) Mean (± SD) Range	52.00 53.72(± 9.41) 24-81		53.50 54.93(± 10.20) 25-83		0.120
BMI median(kg/m^2^) Mean (± SD) Range	24.65 24.99(± 3.66) 16.35-41.87		24.78 25.07(± 3.83) 16.53-39.78		0.800
Hypertension					0.560
No Yes	316 103	75.4 24.6	182 66	73.4 26.6	
Diabetes					0.724
No Yes	359 60	85.7 14.3	210 38	84.7 15.3	
Histologic type					0.357
Type I Type II	290 129	69.2 30.8	180 68	72.6 27.4	
Myometrial invasion					0.627
<1/2 ≥1/2	314 105	74.9 25.1	190 58	76.6 23.4	
Cervical stromal invasion					0.520
No Yes	361 58	86.2 13.8	218 30	87.9 12.1	
Lymphadenectomy					0.552
No Yes	46 373	11.0 89.0	31 217	12.5 87.5	
Adjuvant treatment					0.652
Follow-up or HT Only chemotherapy Only radiotherapy Chemo-radiotherapy	193 164 12 50	46.1 39.1 2.9 11.9	122 96 4 26	49.2 38.7 1.6 10.5	
Ki67 positive ratio (%)					0.707
Median Mean (± SD) Range	30 31.96(± 19.09) 0–90		30 33.30(± 21.81) 0–90		
ER positive ratio (%)					0.507
Median Mean (± SD) Range	90 63.13(± 35.06) 0–95		80 62.39(± 34.19) 0–95		
PR positive ratio (%)					0.136
Median Mean (± SD) Range	85 63.46(± 34.73) 0–95		80 60.28(± 35.69) 0–95		
P53 expression					0.672
Normal Abnormal	221 198	52.7 47.3	135 113	54.4 45.6	
Recurrence					0.901
No Yes	368 51	87.8 12.2	217 31	87.5 12.5	
Death					0.869
No death Death of EC Death of other disease	388 26 5	92.6 6.2 1.2	227 18 3	91.5 7.3 1.2	
Follow-up (months)					0.601
Median Mean (± SD) Range	50.00 49.43 (± 17.37) 12–79		47 48.72 (± 16.08) 14–79		

BMI, Body Mass Index; HT, hormonal treatment; FIGO, International Federation of Gynecology and Obstetrics; Chemo-radiotherapy, chemotherapy and radiotherapy; ER, Estrogen receptor; PR, Progesterone receptor.

**Table 2:** Recurrence characteristics and follow-up.

Variable	Training cohort N = 419	%	Validation cohort N = 248	%	P value
**Recurrence**					
No	368	87.8	217	87.5	0.901
Yes	51	12.2	31	12.5	
**Sites of replased**	51		31		
Vaginal stump	2	3.9	1	3.2	0.977
Central pelvic region	14	27.5	8	25.8	
Lymph nodes (upper para-aortic)	5	9.8	2	6.5	
Peritoneal metastases	11	21.6	8	25.8	
Metastasis to other organs	19	37.3	12	38.7	
**RFS (months)**					
Median	21.00		21.00		0.804
Mean (± SD)	23.02(± 11.89)		24.29 (± 13.70)		
Range	6–55		8–66		
**Follow-up (months)**					
Median	37.00		37.00		0.599
Mean (± SD)	41.16(± 17.01)		39.03(± 17.91)		
Range	13–72		14–74		

RFS, recurrence-free survival.

### Univariate and Multivariate Cox Regression Analysis in Training Cohort

The univariate Cox regression analysis was used to analyze the clinicopathological factors and four immunohistochemical markers (Ki67, ER, PR, P53) that might affect the recurrence of EC (**Table 3**). The factors with P values more than 0.05 were excluded from multivariate analysis, including BMI (P = 0.393), hypertension (P = 0.683), diabetes (P = 0.598), lymphadenectomy (P = 0.890). The other factors with P values less than 0.05, including age, histological type, myometrial invasion, cervical stromal invasion, adjuvant treatment, and all four immunohistochemical markers, were further included in the multivariate Cox regression. Finally, six factors with P values less than 0.05 in multivariate regression analysis were recruited to construct the prediction model, including histological type (P = 0.029), myometrial invasion (P = 0.003), cervical stromal invasion (P = 0.001), Ki67 (P < 0.001), ER (P = 0.009) and P53 expression (P = 0.041).

**Table 3:** Univariate and multivariate analysis of factors predicting EC recurrence in the training cohort.

Variables	Univariate analysis			Multivariate analysis		
	Hazard ratio	95% CI	P-value	Hazard ratio	95% CI	P-value
Age (≥60 vs <60)	1.997	1.147–3.477	0.014	1.124	0.587–2.154	0.724
Histologic type (II vs I)	4.980	2.781–8.915	<0.001	2.233	1.085–4.593	0.029
Myometrial invasion (≥1/2 vs <1/2)	2.516	1.445–4.381	0.001	2.538	1.361–4.733	0.003
Cervical stromal invasion (Yes vs No)	2.653	1.434–4.910	0.002	3.356	1.632–6.902	0.001
Adjuvant treatment (Yes vs No)	2.397	1.296–4.433	0.005	0.465	0.213–1.016	0.055
Ki67 positive ratio (%)	1.043	1.030–1.056	<0.001	1.036	1.020–1.051	<0.001
ER positive ratio (%)	0.977	0.970–0.985	<0.001	0.986	0.975–0.996	0.009
PR positive ratio (%)	0.979	0.971–0.986	<0.001	0.997	0.985–1.008	0.543
P53 expression (Abnormal vs Normal)	2.296	1.283–4.110	0.005	1.902	1.027–3.521	0.041

CI, confidence interval; ER, Estrogen receptor; PR, Progesterone receptor.

### Nomogram Model Establishment and Its Performance

**Figure 2:**
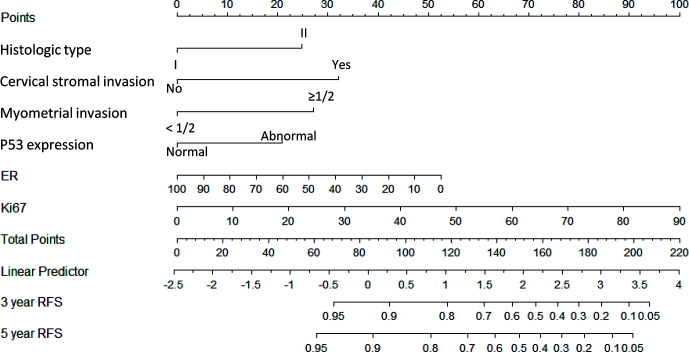
Nomogram model for estimating the rate of recurrence free survival (3 or 5 years) for women with I–II FIGO stage endometrial cancer Description: To estimate the recurrence risk, calculate points for each variable by drawing a straight line from patient’s variable value from the 2nd to 6th line to the 1st line labelled ‘Points’. Sum all points and draw a straight line from the 7th line to the 9th line-10th line to get the 3-, and 5-year recurrence-free survival rate.

**Figure 3:**
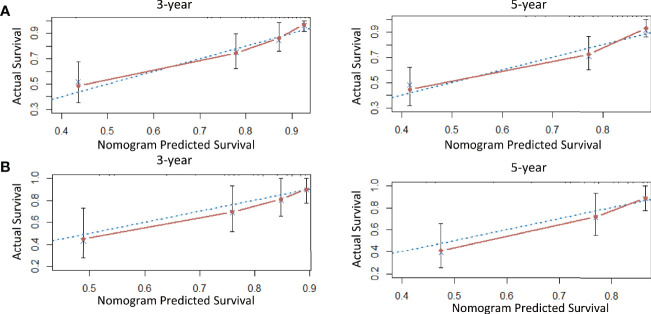
The calibration curve for internal and external validation of the nomogram model. Description: **(A)** The calibration curve for internal validation of the nomogram model for predicting the RFS in EC; **(B)** The calibration curve for external validation of the nomogram model for predicting the RFS in EC (notes: The blue dotted line: Reference line; The red solid line: The prediction curve given by the model).

A nomogram model was established to calculate the RFS in a more convenient and precise manner (**Figure 2**). The weight (prognostic value) of each predictor was given in the nomogram model: the length of each predictor’s line segment represented its own weight, and the score of the weight could be “quantified” by the first “Points” line in the model. It could be seen from the model that the prognostic value of immunohistochemical markers(especially Ki67 and ER) was still very considerable even when compared with classical clinical parameters. The calibration curve showed a good fitness between the prediction and our data in both data sets (**Figure 3**). The current risk stratification systems(RSS), including FIGO classification (25), ESMO classification (15), ESMO/ESGO/ESTRO classification (1), were tested to evaluate their accuracy of prognostic assessment of EC (**Table 4**). Similarly, we also compared the proposed model with those proposed in other similar studies(**Table 5**). For the comparison among different RSS and predicting models, discriminatory power of our proposed model was the highest with the C-index 0.832 (95% CI, 0.752–0.912) in the training cohort; while 0.861 (95% CI, 0.755–0.967) in the validation cohort.

**Table 4:** The discriminatory power (C-index) of different risk stratification systems in the training cohort and validation cohort.

Combinations	Training cohort		Validation cohort	
	C-index	95%CI	C-index	95%CI
FIGO classification	0.645	0.578–0.712	0.646	0.562–0.730
ESMO classification	0.755	0.677–0.833	0.747	0.645–0.849
ESMO-ESGO-ESTRO classification	0.759	0.679–0.839	0.754	0.650–0.858
Our model	0.832	0.752–0.912	0.861	0.755–0.967

CI, confidence interval; FIGO, International Federation of Gynecology and Obstetrics; ESMO, European Society for Medical Oncology; ESMO-ESGO-ESTRO, European Society for Medical Oncology, European Society for Radiotherapy & Oncology and European Society of Gynaecological Oncology.

**Table 5:** The discriminatory power (C-index) of different models.

Model	Author	Criteria	Training cohort	Validation cohort
Model A (26)	Mingzhu Jia et al.	The combined ratio of ER, PR, Ki67, and P53([ER + PR]/[P53 + Ki67]).	0.645	0.646
Model B (6)	Takahashi et al.	Recurrence prediction score(RPS) system composed of age, pathological type, cervical stromal invasion, peritoneal cytology.	0.774	0.618
Model C (4)	Lobna Ouldamer et al.	A nomogram model including age, lymphadenectomy, histologic type, histological grade, lymphovascular space invasion, FIGO staging.	0.82	0.75
Our model	—	A nomogram model including histological type, myometrial invasion, cervical stromal invasion, Ki67, ER and P53 expression.	0.832	0.861

ER, Estrogen receptor; PR, Progesterone receptor; FIGO, International Federation of Gynecology and Obstetrics.

### Optimal Threshold of the Nomogram

**Figure 4:**
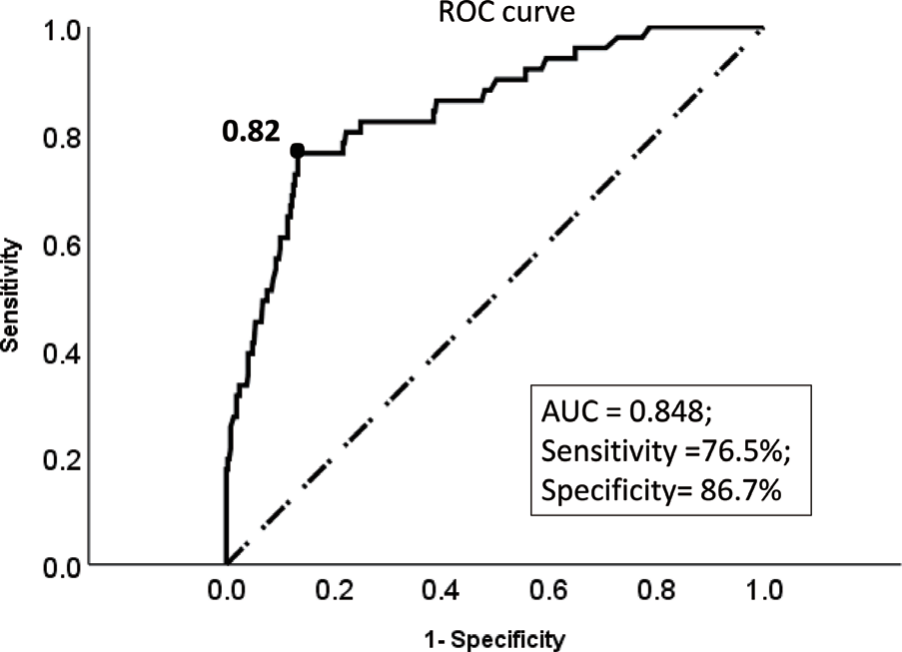
The ROC curve of the optimal threshold value of the 3-year recurrence-free survival rate predicted by the model. Description: Black dot: the area under the curve at this point is the largest, which indicates the optimal threshold value of the 3-year recurrence-free survival rate predicted by the model is 0.82 (area under the curve = 0.848; sensitivity, 76.5%; specificity, 86.7%); (Dotted line: reference line; Solid line: the ROC curve of the model).

**Figure 5:**
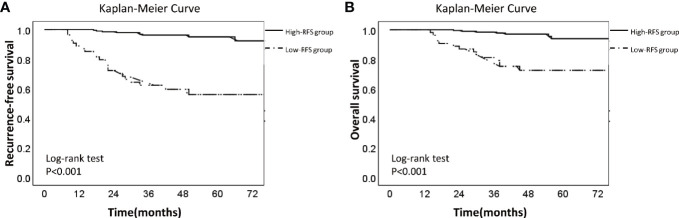
Kaplan-Meier Survival Curve of low-RFS group and high-RFS group. Description: **(A)** Recurrence-free survival curve of low-RFS group and high-RFS group. **(B)** Overall survival curve of low-RFS group and high-RFS group (The dotted line: low-RFS group; The solid line: high-RFS group).

ROC curve showed that the optimal threshold value of the 3-year recurrence-free survival rate predicted by the model was 0.82 (area under the curve = 0.848; sensitivity = 76.5%; specificity = 86.7%) (**Figure 4**). Based on the optimal threshold, patients in the validation cohort with a 3-year recurrence-free survival rate ≥0.82 was defined as high-RFS group, the others were low-RFS group. In the high-RFS group, the median follow-up time and initial time of EC recurrence were 47.5 (range, 22–79) months and 33 (range, 17–66) months; while for the low-RFS group were 45 (range, 14–79) months and 20 (range, 8–50) months, respectively. The 3-year RFS rates for the high-RFS group and low-RFS group were 96.2% (95% CI, 93.5–98.9%) and 62.2% (95% CI, 49.1–75.3%) (P < 0.001). The 3-year overall survival rates of the high-RFS group and low-RFS group were 98.4% (95% CI, 96.6–100%) and 79.1% (95% CI, 68.1–90.1%) (P < 0.001) (**Figure 5**).

## Discussion

Recent years, the Cancer Genome Atlas (TCGA) molecular subgroups of endometrial cancer, including POLE, MSI, copy-number-high, and copy-number-low, showed a good potential prognostic value of endometrial cancer (EC). The prognosis of EC among the four subgroups is significantly different (27–29). But the high cost of genetic test and the high requirements for the detection technology and equipment make it difficult to be used as a common measurement to evaluate the prognosis of EC in many regions. While immunohistochemistry is still an important part of the results of postoperative histopathological survey, as its simplicity, speed and low cost. In our study, we analyzed four immunohistochemical markers (Ki67, ER, PR, and P53) which were commonly used in clinical practice and three (Ki67, ER, and P53) were finally included in our model. With our nomogram model, the postoperative recurrence risk of each patient with FIGO stage I-II EC could be predicted. For example, if a patient with histologic type II (25 points), with cervical stromal invasion (32 points), without deep myometrial invasion (0 points), with P53 expression “normal” (0 points), with ER 40% (+) (32 points) and Ki67 30% (+) (34 points), she would get a total score of 123 points corresponds to a 3-year recurrence-free survival rate of 78% (recurrence probability in 3 years was 22%). Hence our model could explain the abstract concept of postoperative recurrence as a quantitative form based on several predictors instead of a simple conclusion as “high risk” or “low risk”. Meanwhile, through the internal and external verification of calibration curve and the comparison of different models, it was obvious that our model showed a higher prediction accuracy and consistency.

At present, the selection of adjuvant therapy for patients depends on traditional clinicopathological (3). For example, recent European guidelines (1) and NCCN guidelines (30) suggested that supplemental radiotherapy or even combined with chemotherapy may be considered when patients accompany with the following risk factors: late FIGO stage, poor pathological classification, older age (especially >60 yrs), extensive LVSI, and deeper myoinvasion (>50%), etc. However, for the patients with early low-risk endometrial cancer (with stage I and without obvious high-risk factors for recurrence), they usually only received endocrine therapy or follow-up after surgery (30), but unfortunately some of them would still relapse. In our study, the patients in the low-RFS group had poor prognosis compared with the high-RFS group, which indicated that they might be good candidates for adjuvant therapy. Therefore, it might be necessary to pay attention to the postoperative prognosis management of the patients in the low-RFS group: for patients with high-risk factors of recurrence who would receive postoperative adjuvant treatment (chemotherapy and/or radiotherapy), the cycles of the postoperative adjuvant therapy should be increased appropriately compared with the original basis, and the follow-up of them should be more closely; while for some early low-risk EC patients who would only receive endocrine therapy or follow-up after surgery, the appropriate postoperative adjuvant treatment still might be necessary for them. However, there is limited evidence on the clinical benefit of adjuvant chemotherapy for patients with stage I–II endometrial cancer (6). Thus, it is necessary to conduct clinical trials to evaluate the effect of adjuvant chemotherapy on patients in low-RFS group, and our model involving immunohistochemical makers could be used as a reliable tool for risk stratification.

What deserves to be mentioned is that we have to explain the following points: 1) In this study, the immunohistochemical markers ER and Ki67 were incorporated into the nomogram as continuous variables, which were better fit the model compared as the dichotomous variables. Because different positive ratio of each immunohistochemical marker can correspond to a specific score in the model, for example, ER 10% (+) corresponds to “46 points”, ER 80% (+) corresponds to “11 points”, which was more suitable for personalized evaluation of each patient. 2) Particularly, contrary to our impression, according to the risk ratio of the univariate analysis, it seemed that patients who received postoperative adjuvant therapy were more likely to relapse, which might be explained by that most patients who received adjuvant therapy were with late stages, poor types or other high-risk factors of recurrence, it caused adjuvant therapy as a prognostic factor had a strong “collinearity” with these high-risk factors for recurrence in univariate analysis (31), and the “protective effect” of adjuvant therapy was not enough to offset the high-risk of recurrence caused by these risk factors. Therefore the adjuvant treatments were suggested as the “risk factor” in univariate analysis. However, since the collinear effect of these factors was minimized in multivariate analysis, the adjuvant treatments were illustrated as the “protective factor” (HR value of adjuvant treatment < 1 in multivariate analysis). Although the results showed that there was no significant correlation between postoperative adjuvant therapy and recurrence, other studies reported that postoperative adjuvant therapy played a positive role in improving the recurrence-free survival rate of EC patients (32, 33). 3) Finally, the factors such as age, PR, didn’t show the obvious correlation with the recurrence in multivariate analysis, which can’t discredit their importance as EC prognostic factors. In fact, they have been proved to be the important prognosis indicators of endometrial cancer in other reports (1, 34).

Of course, our model could be optimized. Firstly, due to “the early stage” of most endometrial cancer patients when diagnosed and “the low recurrence rate” after surgery, the number of recurrent cases included in the study was relatively small in terms of the number of prognostic factors involved in the multivariate analysis, which may cause statistical bias to a certain extent. So the model requires a larger multi-center data to further verify its universalit. Secondly, many other immunohistochemical markers such as the serum Ca125, the L1 cell adhesion molecule, PTEN have been proved to be related to the prognosis of endometrial cancer in other studies (7, 35, 36), and the molecular classification proposed by TCGA has gained prominence in recent years (27). In future study, it is possible that more immunohistochemical markers and genomic prognostic factors scould be tested and involved in prediction models.

In summary, we have developed a nomogram model to predict the 3-year and 5-year recurrence-free survival of FIGO I-II EC patients. It can be used as a reliable reference tool for patients with stage I–II endometrial cancer who need postoperative adjuvant therapy.

## Data Availability Statement

The original contributions presented in the study are included in the article/**Supplementary Material**. Further inquiries can be directed to the corresponding author.

## Ethics Statement

The studies involving human participants were reviewed and approved by Ethics Committee of Chongqing Medical University. The patients/participants provided their written informed consent to participate in this study.

## Author Contributions

ZYH: conceptualization, methodology, supervision, project administration, and writing (review and editing). PJ: methodology, data curation, investigation, software, formal analysis, writing (original draft preparation), and writing (review and editing). MJ: data curation, data curation, and investigation. JH: data curation and supervision. ZH: data curation and formal analysis. YD: data curation and investigation. All authors contributed to the article and approved the submitted version.

## Conflict of Interest

The authors declare that the research was conducted in the absence of any commercial or financial relationships that could be construed as a potential conflict of interest.

## Abbreviations

EC, endometrial cancer; ER, estrogen receptor; PR, progesterone receptor; LVSI, lymphatic vessel space invasion; RFS, recurrence-free survival; OS, overall survival; FIGO, International Federation of Gynecology and Obstetrics; ROC, Receiver Operating Characteristic; TCGA, The Cancer Genome Atlas.

## References

[1] Colombo N Creutzberg C Amant F Bosse T Gonzalez-Martin A Ledermann J ESMO-ESGO-ESTRO Consensus Conference on Endometrial Cancer: diagnosis, treatment and follow-up. Ann Oncol (2016) 27(1):16–41. 10.1093/annonc/mdv484 26634381

[2] Creasman WT Odicino F Maisonneuve P Quinn MA Beller U Benedet JL Carcinoma of the Corpus Uteri. Int J Gynecol Obstet (2006) 95:S105–43. 10.1016/s0020-7292(06)60031-3 17161155

[3] Francis SR Ager BJ Do OA Huang Y-HJ Soisson AP Dodson MK Recurrent early stage endometrial cancer: Patterns of recurrence and results of salvage therapy. Gynecol Oncol (2019) 154(1):38–44. 10.1016/j.ygyno.2019.04.676 31029507

[4] Ouldamer L Bendifallah S Body G Touboul C Graesslin O Raimond E Predicting poor prognosis recurrence in women with endometrial cancer: a nomogram developed by the FRANCOGYN study group. Br J Cancer (2016) 115(11):1296–303. 10.1038/bjc.2016.337 PMC5129824 27824810

[5] Awtrey CS Nomograms for predicting endometrial cancer recurrence. Gynecol Oncol (2012) 125(3):513–4. 10.1016/j.ygyno.2012.04.035 22608810

[6] Takahashi K Yunokawa M Sasada S Takehara Y Miyasaka N Kato T A novel prediction score for predicting the baseline risk of recurrence of stage I-II endometrial carcinoma. J Gynecol Oncol (2019) 30(1):e8. 10.3802/jgo.2019.30.e8 30479092 PMC6304400

[7] van der Putten LJM Visser NCM van de Vijver K Santacana M Bronsert P Bulten J Added Value of Estrogen Receptor, Progesterone Receptor, and L1 Cell Adhesion Molecule Expression to Histology-Based Endometrial Carcinoma Recurrence Prediction Models: An ENITEC Collaboration Study. Int J Gynecol Cancer (2018) 28(3):514–23. 10.1097/IGC.0000000000001187 29324536

[8] Ohara M Matsuura K Akimoto E Noma M Doi M Nishizaka T Prognostic value of Ki67 and p53 in patients with estrogen receptor-positive and human epidermal growth factor receptor 2-negative breast cancer: Validation of the cut-off value of the Ki67 labeling index as a predictive factor. Mol Clin Oncol (2016) 4(4):648–54. 10.3892/mco.2016.776 PMC4812092 27073684

[9] Abubakar M Figueroa J Ali HR Blows F Lissowska J Caldas C Combined quantitative measures of ER, PR, HER2, and KI67 provide more prognostic information than categorical combinations in luminal breast cancer. Mod Pathol (2019) 32(9):1244–56. 10.1038/s41379-019-0270-4 PMC6731159 30976105

[10] Ferrandina G Ranelletti FO Gallotta V Martinelli E Zannoni GF Gessi M Expression of cyclooxygenase-2 (COX-2), receptors for estrogen (ER), and progesterone (PR), p53, ki67, and neu protein in endometrial cancer. Gynecol Oncol (2005) 98(3):383–9. 10.1016/j.ygyno.2005.04.024 15979129

[11] Gulseren V Kocaer M Ozdemir IA Cakir I Sanci M Gungorduk K Do estrogen, progesterone, P53 and Ki67 receptor ratios determined from curettage materials in endometrioid-type endometrial carcinoma predict lymph node metastasis? Curr Probl Cancer (2020) 44(1):100498. 10.1016/j.currproblcancer.2019.07.003 31395281

[12] McAlpine JN Temkin SM Mackay HJ Endometrial cancer: Not your grandmother’s cancer. Cancer (2016) 122(18):2787–98. 10.1002/cncr.30094 27308732

[13] Pecorelli S Revised FIGO staging for carcinoma of the vulva, cervix, and endometrium. Int J Gynecol Obstet (2009) 105(2):103–4. 10.1016/j.ijgo.2009.02.012 19367689

[14] Marcos-Sanmartin J Lopez Fernandez JA Sanchez-Paya J Pinero-Sanchez OC Roman-Sanchez MJ Quijada-Cazorla MA Does the Type of Surgical Approach and the Use of Uterine Manipulators Influence the Disease-Free Survival and Recurrence Rates in Early-Stage Endometrial Cancer? Int J Gynecol Cancer (2016) 26(9):1722–6. 10.1097/IGC.0000000000000808 PMC5084631 27518143

[15] Preti E Colombo N Landoni F Carinelli S Colombo A Marini C ESMO Guidelines Working Group: Endometrial cancer: ESMO clinical practice guidelines for diagnosis, treatment and follow-up. Ann Oncol (2013) 24(Suppl 6):vi33–8. 10.1093/annonc/mdt353 24078661

[16] Versluis MA de Jong RA Plat A Bosse T Smit VT Mackay H Prediction model for regional or distant recurrence in endometrial cancer based on classical pathological and immunological parameters. Br J Cancer (2015) 113(5):786–93. 10.1038/bjc.2015.268 PMC4559831 26217922

[17] Ouldamer L Bendifallah S Body G Canlorbe G Touboul C Graesslin O Change in hazard rates of recurrence over time following diagnosis of endometrial cancer: An age stratified multicentre study from the FRANCOGYN group. Eur J Surg Oncol (2018) 44(12):1914–20. 10.1016/j.ejso.2018.07.053 30217355

[18] Huijgens ANJ Mertens HJMM Factors predicting recurrent endometrial cancer. FVV ObGyn (2013) 5(3):179–86. PMC3987371 24753943

[19] Yu X Guo S Song W Xiang T Yang C Tao K Estrogen Receptor α (ERα) Status Evaluation Using RNAscope in Situ Hybridization: A Reliable and Complementary Method for IHC in Breast Cancer Tissues. Hum Pathol (2017) 61:121–9. 10.1016/j.humpath.2016.12.005 27993577

[20] Smith D Stewart CJR Clarke EM Lose F Davies C Armes J ER and PR expression and survival after endometrial cancer. Gynecol Oncol (2018) 148(2):258–66. 10.1016/j.ygyno.2017.11.027 29217139

[21] Di Donato V IV Schiavi MC Colagiovanni V Pecorella I Palaia I Perniola G Impact of Hormone Receptor Status and Ki-67 Expression on Disease-Free Survival in Patients Affected by High-risk Endometrial Cancer. Int J Gynecol Cancer (2018) 28(3):505–13. 10.1097/IGC.0000000000001191 29465508

[22] Köbel M RB Singh N Soslow RA Gilks CB McCluggage WG Interpretation of P53 Immunohistochemistry in Endometrial Carcinomas: Toward Increased Reproducibility. Int J Of Gynecol Pathol (2019) 38(Suppl 1):S123–31. 10.1097/PGP.0000000000000488 PMC6127005 29517499

[23] Schisterman EF Perkins NJ Liu A Bondell H Optimal cut-point and its corresponding Youden Index to discriminate individuals using pooled blood samples. Epidemiology (2005) 16(1):73–81. 10.1097/01.ede.0000147512.81966.ba 15613948

[24] Brentnall AR Cuzick J Use of the concordance index for predictors of censored survival data. Stat Methods Med Res (2018) 27(8):2359–73. 10.1177/0962280216680245 PMC6041741 27920368

[25] FIGO Committee on Gynecologic Oncology FIGO staging for carcinoma of the vulva, cervix, and corpus uteri. Int J Gynaecol Obstet (2014) 125(2):97–8. 10.1016/j.ijgo.2014.02.003 24630859

[26] Jia M Jiang P Huang Z Hu J Deng Y Hu Z The combined ratio of estrogen, progesterone, Ki-67, and P53 to predict the recurrence of endometrial cancer. J Surg Oncol (2020) 122(8):1808–14. 10.1002/jso.26212 32920817

[27] Raffone A Travaglino A Mascolo M Carbone L Guida M Insabato L TCGA molecular groups of endometrial cancer: Pooled data about prognosis. Gynecol Oncol (2019) 155(2):374–83. 10.1016/j.ygyno.2019.08.019 31472940

[28] Leon-Castillo A Gilvazquez E Nout R Smit VT McAlpine JN McConechy M Clinicopathological and molecular characterisation of ‘multiple-classifier’ endometrial carcinomas. J Pathol (2020) 250(3):312–22. 10.1002/path.5373 PMC7065184 31829447

[29] Guo S Yang J Wu M Xiao G Clinical value screening, prognostic significance and key pathway identification of miR-204-5p in endometrial carcinoma: A study based on the Cancer Genome Atlas (TCGA), and bioinformatics analysis. Pathol Res Pract (2019) 215(5):1003–11. 10.1016/j.prp.2019.02.007 30910254

[30] Wui-Jin Koh MC F. H. C. Research, C. S. C. C. Alliance, M. Ω. V. C. *Nadeem R. Abu-Rustum, M. S. K. C. Center, M. Sarah Bean, D. C. Institute, M. Kristin Bradley, U. o. Wisconsin, C. C. Center, M. Susana M. Campos, MPH, MS †, D.-F. B. a. Women’s, C. Center, M. Kathleen R. Cho, U. o. Michigan, R. C. Center, M. Ω. Hye Sook Chon, M. C. Center, M. Ω. Christina Chu, F. C. C. Center, R. C. Ω, M. G. Hospital, M. Ω. David Cohn, T. O. S. U. Comprehensive, C. C.-J. C. Hospital, a. S. R. Institute, M. Ω. Marta Ann Crispens, V.-I. C. Center, M. Shari Damast, Y. C. Center/ and S. C. Hospital NCCN Guidelines Version 2.2019 Endometrial Carcinoma. In: NCCN Guidelines for Patients. National Comprehensive Cancer Network (2018). Available at: www.nccn.org.

[31] Pierre-Graud Claret XB de La Coussaye JE Collinearity and multivariable analysis. Intensive Care Med (2016) 42(11):1834. 10.1007/s00134-016-4528-8 27671147

[32] Alessia Aloisi FP Scaletta G Capriglione S Laraud F Miranda A Montera R Chemotherapy as Adjuvant Treatment for Intermediate-High Risk Early-Stage Endometrial Cancer: A Pilot Study. Int J Gynecol Cancer (2015) 25(8):1418–23. 10.1097/IGC.0000000000000505 26186073

[33] Emons G Vordermark, Dirk: Adjuvant treatment for endometrial cancer. Curr Opin Oncol (2019) 31(5):404–10. 10.1097/CCO.0000000000000558 31233482

[34] Violante Di Donato V II Schiavi MC Colagiovanni V Pecorella I Palaia I Perniola G Impact of Hormone Receptor Status and Ki-67 Expression on Disease-Free Survival in Patients Affected by High-risk Endometrial Cancer. Int J Gynecol Cancer (2018) 28(3):505–13. 10.1097/IGC.0000000000001191 29465508

[35] Nakamura K Imafuku N Nishida T Niwa I Joja I Hongo A Measurement of the minimum apparent diffusion coefficient (ADCmin) of the primary tumor and CA125 are predictive of disease recurrence for patients with endometrial cancer. Gynecol Oncol (2012) 124(2):335–9. 10.1016/j.ygyno.2011.10.014 22008707

[36] Raffone A Travaglino A Saccone G Viggiani M Giampaolino P Insabato L PTEN expression in endometrial hyperplasia and risk of cancer: a systematic review and meta-analysis. Arch Gynecol Obstet (2019) 299(6):1511–24. 10.1007/s00404-019-05123-x 30915635

