# Supplementary material for: A Nomogram Model Involving Immunohistochemical Markers for Predicting the Recurrence of Stage I-II Endometrial Cancer
Source: Front Oncol. 2021 Jan 22;10:586081. doi: 10.3389/fonc.2020.586081 (PMC7874072; doi:10.3389/fonc.2020.586081)
Supplement: Supplementary file 1 [file Table_1.docx]

**APPENDIX**

**Related Computerized Programs for Nomogram With R**

library(rms)

**For dividing data sets into training cohort and vilidation cohort**

library(caret)

library(rms)

alldata_credit <- read.csv("f:\\alldata_credit.csv")

train <-createDataPartition(y=alldata_credit$Figo,p=0.70,list=FALSE)

traindata <- alldata_credit[train, ]

testdata <- alldata_credit[-train, ]

**For Nomogram**

library(survival)

library(rms)

data(package="survival")

dd<-datadist(traindata)

options(datadist="dd")

f<-cph(Surv(traindata$time,traindata$recurrence)~histologicaltype+myometrialinvasion+cervicalstromalinvasion+Ki67+ER+P53,data=traindata,x=TRUE,y=TRUE,surv=TRUE)

survival<-survival(f)

survival1<-function(x)survival(12,x)

survival2<-function(x)survival(36,x)

survival3<-function(x)survival(60,x)

nom<-nomogram(f,fun=list(survival1,survival2,survival3),fun.at = c(0.1,seq(0.2,0.8,by=0.1),0.9),funlabel = c('1 year RFS','3 year RFS','5 year RFS'))

plot(nom)

**For Computing the C-Index and 95% CI in training cohort and vilidation cohort**

library(survival)

library(rms)

fit<-coxph(Surv(time,recurrence)~ histologicaltype+myometrialinvasion+cervicalstromalinvasion+Ki67+ER+P53,data = traindata)

survConcordance(Surv(traindata$DST,traindata$recurrence)~predict(fit,traindata))

fit<-coxph(Surv(time,recurrence)~histologicaltype+myometrialinvasion+cervicalstromalinvasion+Ki67+ER+P53,data = traindata)

survConcordance(Surv(testdata$time,testdata$recurrence)~predict(fit,testdata))

**For predictions of the vilidation cohort**

library(survival)

library(rms)

f<-cph(Surv(time,recurrence)~histologicaltype+myometrialinvasion+cervicalstromalinvasion+Ki67+ER+P53,data = traindata)

fp<-predict(f,newdata = testdata)

predictions<-predict(f,newdata = testdata)

predictions

**For Calibration Curve**

library(survival)

library(rms)

f1<-cph(Surv(traindata$time,traindata$recurrence,type = "right")~ histologicaltype+myometrialinvasion+cervicalstromalinvasion+Ki67+ER+P53,data = traindata,x=TRUE,y=TRUE,surv=TRUE,time.inc = 3*12)

cal=calibrate(f1,cmethod = 'KM',method = "boot",u=3*12,m=30,B=1000)

plot(cal,lwd=1,lty=1,errbar.col=c(rgb(0,0,0,maxColorValue = 255)),xlim = c(0,1),ylim = c(0,1),xlab ="Nomogram Predicted Survival",ylab="Actual Survival",col=c(rgb(255,0,0,maxColorValue =255)))

abline(0,1,lty = 3,lwd = 2,col = c(rgb(0,118,192,maxColorValue=255)))

lines(cal[,c('mean.predicted','KM')], type = 'b',lwd = 2, col = c(rgb(192,98,83,maxColorValue = 255)),pch = 16)

**For External Validation of Nomogram**

f2<-cph(Surv(testdata$time,testdata$recurrence,type = "right")~predictions,x=T,y=T,surv=T, time.inc =3*12)

validate(f2,method = "boot",B=1000,dxy=T,u=3*12)

**For Calibration Curve for Validation Cohort**

cal<-calibrate(f2,cmethod = 'KM',method = "boot",u=3*12,m=20,B=1000)

plot(cal,lwd=1,lty=1,errbar.col=c(rgb(0,0,0,maxColorValue = 255)),xlim = c(0,1),ylim = c(0,1),xlab ="Nomogram Predicted Survival",ylab="Actual Survival",col=c(rgb(255,0,0,maxColorValue =255)))

abline(0,1,lty = 3,lwd = 2,col = c(rgb(0,118,192,maxColorValue=255)))

lines(cal[,c('mean.predicted','KM')], type = 'b',lwd = 2, col = c(rgb(192,98,83,maxColorValue = 255)),pch = 16)
